# Coexistence of restless legs syndrome and multiple sclerosis aggravates anxiety and depression

**DOI:** 10.1590/0004-282X-ANP-2020-0400

**Published:** 2022-02-06

**Authors:** Serhan SEVIM, Meltem DEMIRKIRAN, Murat TERZI, Nur YÜCEYAR, Bahar TAŞDELEN, Egemen İDIMAN, Murat KÜRTÜNCÜ, Cavit BOZ, Deniz TUNCEL, Rana KARABUDAK, Aksel SIVA, Abdülcemal ÖZCAN, Münife NEYAL, Başak Karakurum GÖKSEL, Gülcan Baran GAZALOĞLU, Mehmet BALAL, Sedat ŞEN, Meltem Alkaya BAKLAN, Tuncay GÜNDÜZ, Aslı TUNCER, Uğur UYGUNOĞLU

**Affiliations:** 1 Mersin University, School of Medicine, Department of Neurology, Mersin, Turkey. Mersin University School of Medicine Department of Neurology Mersin Turkey; 2 Çukurova University, Department of Neurology, Adana, Turkey. Çukurova University Department of Neurology Adana Turkey; 3 Ondokuz Mayıs University, Department of Neurology, Samsun, Turkey. Ondokuz Mayıs University Department of Neurology Samsun Turkey; 4 Ege University, Department of Neurology, İzmir, Turkey. Ege University Department of Neurology İzmir Turkey; 5 Mersin University, Department of Biostatistics, Mersin, Turkey. Mersin University Department of Biostatistics Mersin Turkey; 6 Dokuz Eylül University, Department of Neurology, İzmir, Turkey. Dokuz Eylül University Department of Neurology İzmir Turkey; 7 İstanbul University, Department of Neurology, İstanbul, Turkey. İstanbul University Department of Neurology İstanbul Turkey; 8 Karadeniz Technical University, Department of Neurology, Trabzon, Turkey. Karadeniz Technical University Department of Neurology Trabzon Turkey; 9 Sütçü İmam University, Department of Neurology, Kahramanmaraş, Turkey. Sütçü İmam University Department of Neurology Kahramanmaraş Turkey; 10 Hacettepe University, Department of Neurology, Ankara, Turkey. Hacettepe University Department of Neurology Ankara Turkey; 11 İstanbul University, Cerrahpaşa School of Medicine, Department of Neurology, İstanbul, Turkey. İstanbul University Cerrahpaşa School of Medicine Department of Neurology İstanbul Turkey; 12 İnönü University, Department of Neurology, Malatya, Turkey. İnönü University Department of Neurology Malatya Turkey; 13 Sanko University, Department of Neurology, Gaziantep, Turkey. Sanko University Department of Neurology Gaziantep Turkey; 14 Başkent University, Department of Neurology, Adana, Turkey. Başkent University Department of Neurology Adana Turkey; 15 Mersin University, Department of Neurology, Mersin, Turkey. Mersin University Department of Neurology Mersin Turkey

**Keywords:** Multiple Sclerosis, Restless Legs Syndrome, Depression, Anxiety, Mental Disorders, Esclerose Múltipla, Síndrome das Pernas Inquietas, Depressão, Ansiedade, Transtornos Mentais

## Abstract

**Background:**

Among the comorbidities that accompany multiple sclerosis (MS), restless legs syndrome (RLS) is one of the most common. Anxiety and depression are common psychological comorbidities that impact the quality of life of patients with MS (PwMS), as well as patients with RLS.

**Objective:**

To investigate the psychiatric burden of MS and RLS coexistence, we conducted a nationwide, multicenter and cross-sectional survey.

**Methods:**

Participants were assessed by using demographic and clinical parameters along with the Hamilton Anxiety and Hamilton Depression Scales (HAM-A and HAM-D).

**Results:**

Out of the 1,068 participants, 173 (16.2%) were found to have RLS [RLS(+)] and 895 (83.8%) did not [RLS(-)]. The mean scores for HAM-A and HAM-D were significantly higher among RLS(+) subjects than among RLS(-) subjects (p<0.001 for all variables).

**Conclusions:**

According to our data, the presence of RLS in PwMS may increase the occurrence of both anxiety and depression symptoms. Awareness and treatment of RLS in PwMS could possibly reduce the symptoms of psychiatric comorbidities originating from RLS.

## RESUMO

**Antecedentes:**

Considerando-se as comorbidades que acompanham a esclerose múltipla (EM), a síndrome das pernas inquietas (SPI) é uma das mais comuns, e ansiedade e depressão são comorbidades psicológicas comuns que afetam a qualidade de vida de pacientes com EM, bem como de pacientes com SPI.

**Objetivo:**

Investigar a carga psiquiátrica da coexistência de EM e SPI por meio de uma pesquisa nacional, multicêntrica e transversal.

**Métodos:**

Os participantes foram avaliados por parâmetros demográficos e clínicos, além da versão turca das escalas de ansiedade e depressão de Hamilton (HAM-A e HAM-D).

**Resultados:**

Dos 1.068 participantes, 173 (16,2%) apresentaram SPI [SPI (+)] e 895 (83,8%) não [SPI (-)]. As pontuações médias no HAM-A e no HAM-D foram significativamente maiores em indivíduos com SPI (+) do que naqueles com SPI (-) (p <0,001 para todas as variáveis).

**Conclusões:**

De acordo com nossos dados, a presença de SPI na EM pode aumentar a ocorrência de sintomas de ansiedade e depressão. A conscientização e o tratamento da SPI na EM podem reduzir os sintomas de comorbidades psiquiátricas originadas da SPI.

## INTRODUCTION

Multiple sclerosis (MS) has been reported to be associated with many autoimmune, physical and psychiatric conditions. Among the comorbidities that accompany MS, restless legs syndrome (RLS) is one of the most common, and it is probably the only condition that has been shown to be three to fivefold more prevalent among patients with MS (PwMS) than among the general population, in the relevant case-control studies^1^^,^^2^^,^^3^^,^^4^. Moreover, anxiety and depression are common psychological comorbidities that impact the quality of life and even increase suicidal tendencies among patients with multiple sclerosis (PwMS), as well as among patients with RLS^5^^,^^6^^,^^7^^,^^8^^,^^9^^,^^10^. Nonetheless, except for two very recent studies, there has not been much effort to show how the prevalence of RLS affects both depression and anxiety among PwMS^10^^,^^11^^,^^12^.

We aimed to investigate the interaction between anxiety/depression and RLS among PwMS in a nationwide, multicenter and cross-sectional survey. The survey included demographic, clinical and biochemical variables. Exploring the relations of some of these variables with anxiety and depression among PwMS who had coexisting RLS could possibly contribute towards understanding the causes of the high prevalence rates of this psychiatric burden and the pathophysiology of both diseases.

## METHODS

Data were drawn from the ‘Restless Legs of Multiple Sclerosis -Turkey’ project (RELOMS-T), which had the objective of examining the consequences of coexistence of MS and RLS and was designed to represent all PwMS throughout Turkey^13^. Patients who were 18 years or older and had a diagnosis of MS or clinically isolated syndrome (CIS) in accordance with the revised 2010 version of the McDonald criteria were recruited at the MS centers of 13 university hospitals^14^.

### Estimation of sample size

The study was planned to represent all PwMS throughout the country. Considering the population of Turkey (80 million) and the results from MS prevalence studies that have been conducted in this country, the prevalence rate of MS is estimated to be 60/100,000 and the total number of PwMS throughout the country, 45,000. Assuming that the prevalence of RLS among PwMS was 20%, with a 95% confidence interval, an error of 3% and a total number of PwMS of 45,000, the minimum sample size was calculated to be 942 patients (Raosoft, Inc., 2004).

### Study procedure

The survey consisted of face-to-face interviews with 1,089 PwMS. All the interviews were conducted by a neurologist with experience in the field of MS. A three-part questionnaire that asked about the following was used: demographic features, consisting of 8 items (age, gender, sociodemographics, height, weight and smoking status); clinical characteristics, consisting of 15 main items of MS; and lastly, the main subject of this paper, i.e. the Turkish version of the HAM-A and HAM-D, to explore the psychiatric burden of MS and RLS coexistence. This questionnaire was applied to each patient included in the study. The following patients were excluded: those under the age of 18; pregnant women; those with a diagnosis of diabetes or uremia; those with a MS relapse within 3 months from the time of interview; those who were receiving any drug used to treat RLS, including dopamine agonists, dopaminergic agents, antiepileptics such as pregabalin, gabapentin and carbamazepine, benzodiazepines and opiates for another disorder or another consequence of MS other than RLS; and those who were unable to answer the questions of the survey.

The Turkish version of the five criteria suggested by the International Restless Legs Syndrome Study Group (IRLSSG) was used in the assessment of RLS^15^: 1) an urge to move legs, usually accompanied with unpleasant sensations; 2) worsening of these unpleasant sensations at rest; 3) partial or complete relief of the urge through movement; 4) an urge to move the legs and accompanying unpleasant sensations that only occurred in the evening or night or was worse at these times than during the day; and 5) an urge to move the legs and accompanying unpleasant sensations that were not solely accounted for as symptoms primary to another medical or behavioral condition. Only those who were found to fulfil all of these five criteria were classified as having RLS at predigit level. The interviewers conducted detailed neurological examinations to distinguish RLS from other disorders that can mimic RLS (particularly symptoms and signs of lower limb impairment due to MS), in cases of necessity.

The interview followed a two-part questionnaire for screened-positive patients who were not currently being treated for RLS. The objective of the structured first part (8 structured items) was to determine the characteristics of RLS. The second part comprised the validated Turkish form of the IRLSSG rating scale and was used to measure the severity of RLS^16^.

Before interviewing each patient, written informed consent was obtained. This study was approved by the Institutional Review Board of Mersin University, and it was conducted in accordance with the Declaration of Helsinki.

### Statistical analysis

All the data were coded using the Stata Data Analysis and Statistical Software (version 15, Stata Corp LLC) data entry program and were summarized using frequency and contingency tables for categorical variables and means and standard deviation for continuous variables. The prevalence of RLS among PwMS was estimated through descriptive statistics. We used the chi-square test to test the independence of the classification criteria and an independent Student’s *t*-test to compare the means. The Mann-Whitney U test was used to compare the mean ranks between the groups and the z test to compare proportions. The three questions about sleep disorders in the HAM-D and one in the HAM-A were excluded from the comparisons between RLS(+) and RLS(-) patients, to avoid bias in the scores for sleep-related questions, which are expected to be much higher in RLS(+) and hence to improve the power of the data. P values <0.05 were regarded as significant.

## RESULTS

**Figure 1.**
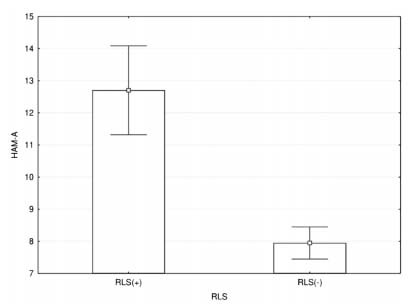
Anxiety scores on HAM-A among RLS(+) and RLS(-) patients. The mean HAM-A score of RLS(+) patients with multiple sclerosis was significantly higher than that of patients with multiple sclerosis without restless legs syndrome (p<0.001).

**Figure 2.**
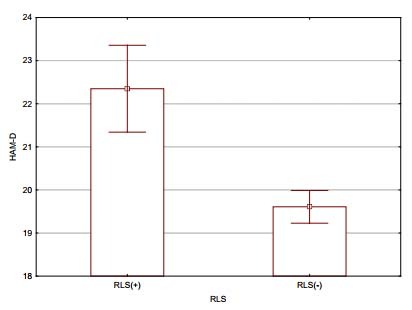
Depression scores on HAM-D among RLS(+) and RLS(-) patients. The mean HAM-D score of RLS(+) patients with multiple sclerosis was significantly higher than that of patients with multiple sclerosis without restless legs syndrome (p<0.001).

In total, 1,089 completed questionnaire forms were collected from 13 centers. Due to uncertainty regarding some demographic data, 21 patients were excluded from the study and hence 1,068 patients were enrolled. Out of the 1,068 patients, 173 (16.2%) were found to be screen-positive for RLS [RLS(+)] according to the diagnostic criteria of the IRLSSG (128 women and 45 men), while 895 (83.8%) were not [RLS(-)]. The mean age of the RLS(+) patients was 38.9 years (SS: 9.7; min: 18; max: 76). Among the 173 RLS(+) patients, all but 8 of them (4.6%) were underdiagnosed in terms of RLS. Among these 165, the mean IRLSSG rating scale score was 21.4 (SD: 6.6; min: 6, max: 37) and the impact of RLS was mild in 7 (4.2%), moderate in 62 (37.6%), severe in 80 (48.5%) and very severe in 16 (9.7%). The mean age and severity of MS among RLS(+) and RLS(-) patients were compatible. Comparisons of some of the demographic and clinical characteristics of RLS(+) and RLS(-) patients are shown in Table 1. The mean (±SD) anxiety scores of RLS(+) and RLS(-) patients were 12.7 (SD: 4.5) and 7.9 (SD: 3.5) respectively; and depression 22.4 (SD: 6.8) and 19.6 (SD: 5,8) The mean scores for HAM-A and HAM-D and the two subscales of HAM-A assessing psychic and somatic functioning were found to be significantly higher among RLS(+) subjects than among the RLS(-) subjects (p<0.001 for all variables) (Figures 1 and 2).

**Table 1.** Comparison of some of the demographic and clinical characteristics of RLS(+) and RLS(-) patients.

		RLS(+) patients; n=173	RLS(+) patients; n=897	p-value
Characteristics	Age (SD), y	38.9 (9.7)	37.3 (10.4)	0.058
	No. of women (%)	133 (76.9)	664 (74.1)	0.974
	BMI (SD)	25.8 (4.7)	25.8 (5.2)	0.999
	Smokers, no. (%)	64 (37)	201 (22.5)	0.001*
Clinical MS type	CIS (%)	9 (5.2)	65 (7.3)	0.320
	RRMS (%)	150 (86.7)	730 (81.6)	0.107
	SPMS (%)	3 (1.7)	70 (7.8)	0.004*
	PPMS (%)	11 (6.7)	30 (3.4)	0.041*
	Overall (%)	173 (100)	895 (100)	
	EDSS (SD)	1.98 (1.42)	1.98 (1.61)	1.000

Age: mean age of the groups; SD: standard deviation; BMI: mean body-mass index; Smokers: no. of smokers in the groups; CIS: clinically isolated syndrome; RRMS: relapsing-remitting multiple sclerosis; SPMS: secondary progressive multiple sclerosis; PPMS: primary progressive multiple sclerosis; EDSS: Expanded Disability Status Scale; *indicates a statistically significant p-value.

## DISCUSSION

Symptoms of both diseases (MS and RLS) may cause distress and lead to psychiatric illness and a decreased sense of wellbeing. Moreover, very recently, both conditions were shown to trigger suicidal behavior independently, even in the absence of depression^7^^,^^9^^,^^12^. The data on the psychiatric aspects of this coexistence are insufficient, as also are the data on the relationship between the extent of the symptom burden, the risk of psychological symptoms and the beneficial effects of accurate treatment of RLS in PwMS with regard to any psychiatric disorder accompanying this coexistence.

Our survey was the first nationwide multicenter study, involving more than 1,000 patients, to investigate the interaction between psychiatric symptoms and RLS among PwMS. We investigated the effects of coexistence of these two neurological conditions with depression and anxiety as well as comparing their prevalence with each other. According to our data, the presence of RLS in PwMS may increase the occurrence of both anxiety and depression symptoms.

Our findings were compatible with two very recent studies in which the researchers used self-rated depression and anxiety scales and found higher anxiety/depression and lower quality of life (QoL) scores in their RLS(+) PwMS, compared with RLS(-)^10^^,^^11^.

This study had its limitations. The most important limitation of our study was its inability to definitely determine and exclude the effect of other risk factors for depression and anxiety, other than RLS. We did not perform correlation analyses to eliminate the possible triggering effect of other MS symptoms and other risk factors for depression and anxiety, such as poor diet, lack of exercise, high blood sugar levels and hormone imbalances, on depression and anxiety symptoms. In this regard, although there were no differences in age and disease severity between our RLS(+) and RLS(-) groups, the data from this survey cannot be as reliable as the data from studies that are specifically designed to determine the psychiatric burden of MS or RLS and which used correlation analyses. Moreover, we did not assess the quality-of-life of patients and their level of fatigue, which have both been shown to be related to affective disorders. Comparison of patients’ quality of life and fatigue measures with their psychiatric burdens would probably provide greater support for us to comment more precisely on the augmentation effect of RLS on the psychiatric symptoms of PwMS.

On the other hand, the strengths of this study lie in its design of face-to-face interviews conducted by experienced neurologists in this field, the very high number of PwMS included in the study and the power of this study to represent all PwMS throughout the country.

In conclusion, according to our data, coexistence of RLS has an additional effect on the psychiatric burden of PwMS. Although RLS mostly impairs quality of life, it is a treatable condition when recognized. Treatment of RLS in PwMS could possibly reduce the symptoms of psychiatric comorbidities originating from RLS. Thus, it is important to heighten the awareness of this comorbidity and its psychiatric burden among physicians, in order to improve the quality of life of PwMS.
